# Atypical clinical presentation of mucopolysaccharidosis type II (Hunter syndrome): a case report

**DOI:** 10.1186/1752-1947-4-154

**Published:** 2010-05-26

**Authors:** Gauri Shankar Shah, Tania Mahal, Subodh Sharma

**Affiliations:** 1 Department of Pediatrics and Adolescent Medicine, B P Koirala Institute of Health Sciences, Dharan, Nepal

## Abstract

**Introduction:**

We present a very rare case of mucopolysaccharidosis with atypical presentation such as mild mental retardation, an acrocephalic head and no corneal clouding. The purpose of presenting this case is to highlight the distinctive manifestation of mucopolysaccharidosis type II (Hunter syndrome).

**Case presentation:**

A 10-year-old East Asian boy presented with abdominal distension of five years' duration and complained of shortness of breath on and off for the same period. On examination his head was large and his head circumference was 54.5 cm. His neck was short, he had coarse facial features, a depressed nasal bridge and small stubby fingers with flexion of distal interphalangeal joints, and a low arched palate was observed. There was mild mental retardation.

**Conclusion:**

Based on clinical findings and radiological features it is possible to diagnose a case of mucopolysaccharidosis.

Careful and systemic approach is needed to accurately diagnose the exact type as enzymatic studies are not available in most centers.

## Introduction

Mucopolysaccharidosis (MPS) is a group of autosomal recessive metabolic disorders caused by the absence or malfunctioning of the lysosomal enzymes needed to break down molecules called glycosaminoglycans (GAGs). These are long chains of sugar carbohydrates in each cell that help build bone, cartilage, tendons, corneas, skin and connective tissues. Glycosaminoglycans (formerly called mucopolysaccharides) are also found in the fluid that lubricates joints. People with MPS either do not produce enough of one of the 11 enzymes required to break down these sugar chains into proteins and simpler molecules, or they produce enzymes that do not work properly. Over time, these GAGs collect in the cells, blood and connective tissues. This results in permanent, progressive cellular damage which affects the appearance, physical abilities, organ and system functioning and, in most cases, mental development. Common clinical presentation includes facial dysmorphism, hepatosplenomegaly, joint stiffness and contractures, pulmonary dysfuction, myocardial enlargement and valvular dysfunction and neurological involvement. As there is no effective therapy for MPS type II (Hunter syndrome), care has been predominantly palliative. However, enzyme replacement therapy (ERT) with recombinant human iduronate-2-sulfatase has now been introduced. We report this case of MPS type II because of its rarity and the atypical features of mild mental retardation with normal intelligence, acrocephalic head, no corneal clouding and all other features suggestive of MPS type II. Therefore, not all MPS necessarily show symptoms of mental retardation, corneal clouding or atypical features such as a dolichocephalic head. The purpose of presenting this case is to highlight the distinctive manifestation of Hunter syndrome.

## Case presentation

A ten-year-old East Asian boy presented to the Pediatric Out-Patients Department with abdominal distension of five years' duration and having suffered shortness of breath on and off for the same period. Abdominal distension, without abdominal pain, had been gradual and progressive since he was five. There was also a history of joint pain on and off during the past two to three years. There was no history of constipation, diarrhea, vomiting, bleeding, jaundice, seizure, weight loss or loss of appetite or of consciousness. His bladder habit was normal. He started school at seven years of age and was below average in his studies.

**Figure 1:**
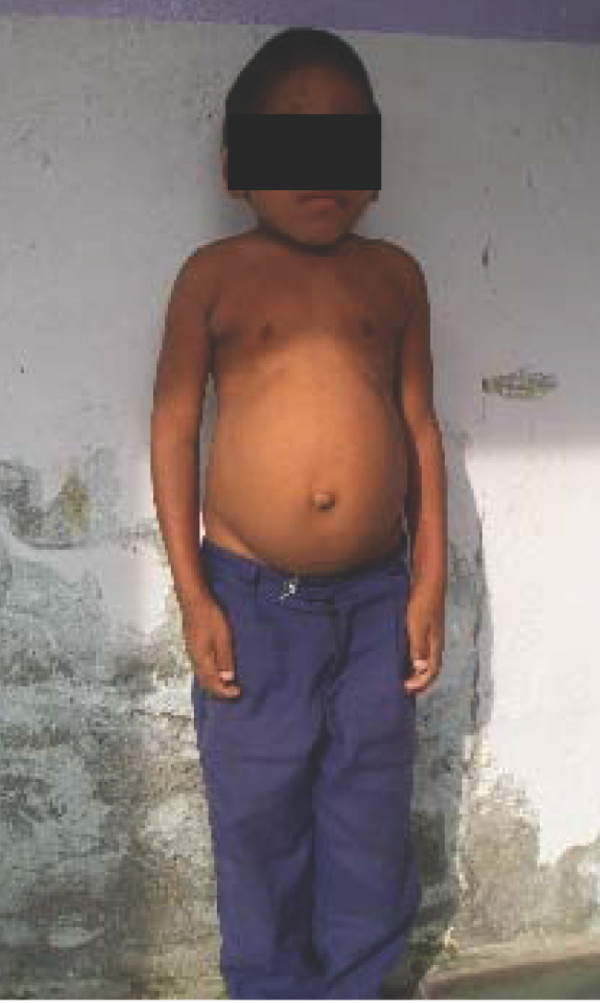
**Child with mucopolysaccharidosis showing an acrocephalic head, coarse facial features, a depressed nasal bridge and a protuberant abdomen**.

**Figure 2:**
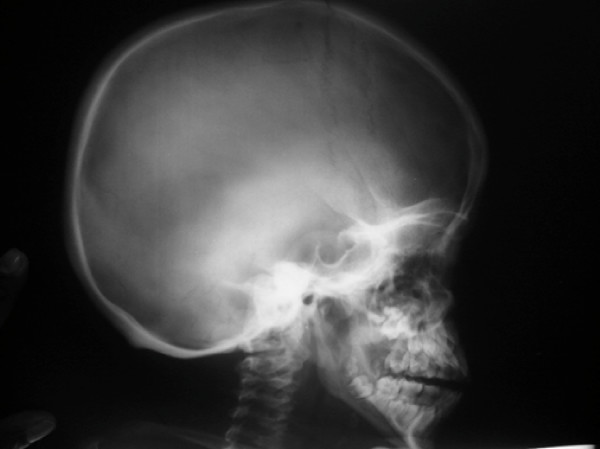
**Anteroposterior and lateral X-ray of the skull showing a 'J' shaped sella turcica**.

**Figure 3:**
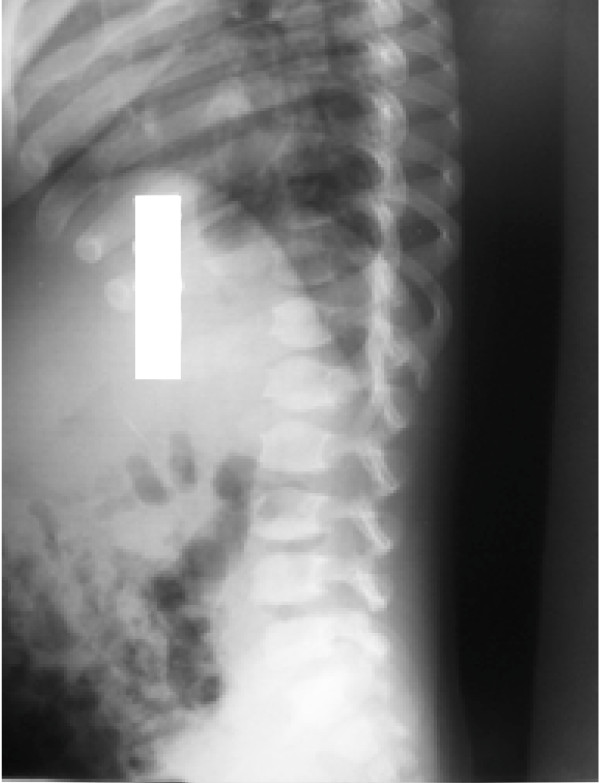
**Anteroposterior and lateral view of the dorsolumbar spine showing beaking of vertebrae**.

**Figure 4:**
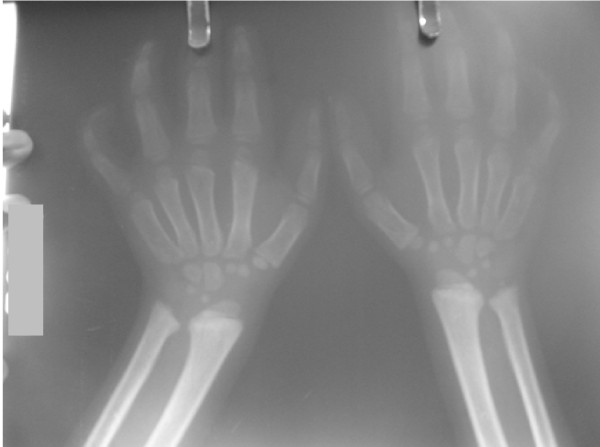
**X-ray of the hands showing phalanges and metacarpals are widened with proximal tapering of metacarpals**.

**Figure 5:**
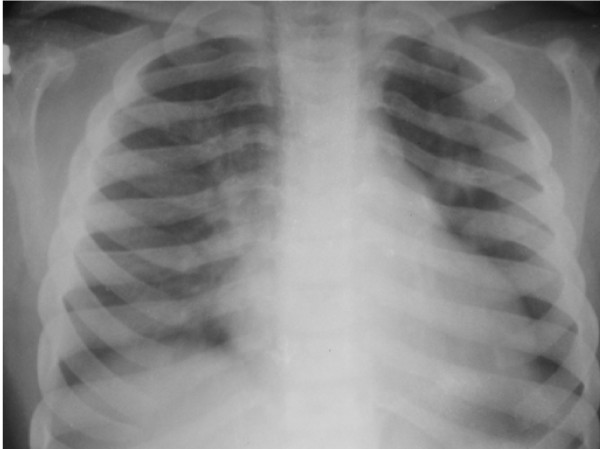
**Anteroposterior chest X-ray showing paddle and/or spatulated ribs**.

On examination his head was acrocephalic in shape, with a circumference of 54.5 cm. He had a depressed nasal bridge, a short neck, coarse facial features, small stubby fingers with flexion of the distal interphalangeal joint, and a low arched palate (Figure 1). Anthropometric examination showed him to be severely stunted without wasting. His fundus appeared normal. His abdomen was soft and slightly distended with a protruding umbilicus. His liver was 11 cm below the right costal margin in the mid-clavicular line, with a firm, sharp margin and a smooth surface with a span of 12 cm. His spleen was 2 cm below the left costal margin in the mid-clavicular line. His heart had a grade 2/6 non-radiating systolic murmur in the mitral area. Suspecting MPS, we performed a skeletal survey. Anteroposterior and lateral X-rays of the skull showed an enlarged and J-shaped sella turcica (Figure 2). The bones of the skull and sutures appeared normal for his age. Anteroposterior and lateral X-rays of the dorsolumbar spine showed anterior beaking (Figure 3). Vertebral bodies appeared ovoid due to convexity of the superior and inferior surfaces. Spinal curvature was normal and vertebral body height also appeared normal. X-rays of both hands showed his phalanges and metacarpals to be widened with proximal tapering of the metacarpals (Figure 4). The remaining bones and joints under view appeared normal. An anteroposterior X-ray showed his ribs as wide with tapered posterior ends (a paddle and/or spatulated appearance) (Figure 5). No parenchymal lesions were seen in visualized lung fluids.

Although his radiological features were suggestive of MPS, without determining the type of MPS, from his history and clinical examination we have made a diagnosis of MPS type II (Hunter syndrome). However in our patient there was atypical presentation such as an acrocephalic head, mild mental retardation, and no corneal clouding, which are important features of MPS type II. Unfortunately we could not perform any measurement of GAG, keratan and heparan sulphates, in his urine because of the lack of test kits. Enzyme assay for iduronate sulfatase is not carried out in our laboratory therefore it was not performed either. Our diagnosis of MPS was confirmed from his history, clinical examination and skeletal survey.

## Discussion

In our case all the clinical features such as short stature, a large head, organomegaly, a depressed nasal bridge, a short neck, coarse facial features, small stubby fingers, mild mental retardation with normal intelligence; and radiological features such as a J-shaped sella turcica, beaking of vertebrae, proximal tapering of metacarpal bones and tapering of the posterior ends of ribs (paddle and/or spatulated ribs) as well as his developmental history were all suggestive of MPS type II.

Mucopolysaccharidosis was first described by Charles Hunter, a Canadian physician, who in 1917 described a rare disease found in two brothers [1,2]. Mucopolysaccharidosis is a group of inherited diseases characterized by defective lysosomal enzymes responsible for the degradation of mucopolysaccharides, which are major components of intercellular connective tissue. This leads to an accumulation of incompletely degraded mucopolysaccharides in the lysosomes which affect various body systems through enzymatic activity [3]. All MPS are autosomal recessive, except Hunter syndrome which is X-linked recessive. In affected individuals, undegraded or partially degraded GAG accumulates within the lysosomes and is excreted in excess in the urine. The accumulation of GAG within the lysosomes is responsible for the clinical manifestation of this disorder [4].

Mucopolysaccharidosis type II or Hunter syndrome is rare and is caused by a deficiency of iduronate-2-sulfatase. Hunter syndrome is one of the most common MPS with a prevalence of one in 170,000 male live births. MPS type II is classified into mild (type II, HB) and severe (type II, A) and this classification is based on the length of survival and the presence or absence of central nervous system (CNS) disease. Patients typically appear normal at birth in both types. In the severe form the clinical features appear between two and four years of age while in the mild form the clinical features appear in the second decade of life. In the severe form there is severe mental retardation and loss of skills. Death usually occurs in the first or second decade of life and the main cause of death is obstructive airway disease or cardiac failure. In the milder form there is mild mental retardation but intelligence is normal, stature is near normal, and clinical features are less obvious and progress very slowly. Diagnosis is usually made in the second decade of life. Death usually occurs in the fourth decade and the main cause of death is cardiac failure.

Diagnosis of the disease is usually made by clinical presentation and skeletal survey. The common clinical presentations are a large head (dolichocephalic), short stature, mental retardation, coarse facial features, a protuberant abdomen, a broad nose with flared nostrils, large jaws, hypotonia and a large tongue which becomes apparent between two and four years of age, and these clinical features were present in our case. Other clinical features include upper respiratory tract infection, valvular heart disease leading to right and left ventricular hypertrophy and heart failure, chronic diarrhea, enlarged liver and spleen, umbilical as well as inguinal hernia, corneal clouding with poor vision and hearing loss caused by both connective and sensorineural deficits. A communicating hydrocephalus is a common finding and can lead to severe manifestation of neurological signs which were not present in our case.

Analysis of GAGs (heparan and dermatan sulphates) is a screening test for MPS type II. The presence of excess heparan and dermatan sulphates in the urine is evidence of MPS type I, MPS type II or MPS type VII. Confirmatory diagnosis is by enzyme assay in leukocytes, fibroblasts or dried blood spots and plasma sample, using substrates specific for 12S. Absent or low 12S activity in males is diagnostic of Hunter syndrome, provided other sulfatase deficiency has been ruled out.

Enzyme replacement therapy using idursulfase (Elaprase), a recombinant human 12S produced in the human cell line, has been recently approved in the United States and the European Union for the management of MPS type II. Weekly intravenous infusion is given over three hours at a dose of 0.5 mg/kg diluted in saline. Bone marrow transplantation (BMT) and umbilical cord blood transplantation (UCBT) are definitive treatments for MPS. Apart from these, supportive management is very important. Physical therapy and daily exercise may improve mobility of joints. Blood transfusion, infection and nutritional management are also important in the management of MPS type II. It has been found that fibroblast from patients shows metachromatic cytoplasmic inclusions [5]. A mild form (MPS type IIB) is compatible with survival into adulthood, and reproduction is known to have occurred [6]. Six cases of this mild form of Hunter syndrome have been described, and the patients survived to the ages of 65 and 87 in two cases. Three of these affected men had children [7]. The enzyme deficient in this disorder is iduronate sulfatase, as described by Neufeld (1987) [8]. Occurrence of Mongolian spots in seven Japanese infants with Hunter syndrome before and after hematopoietic stem cell transplantation (HSCT) has been observed [9].

## Conclusion

Mucopolysaccharidosis is a multisystem disorder which presents with a constellation of clinical findings. Careful and systemic approach is needed to accurately diagnose the exact type as enzymatic studies are not available in most centers.

## Consent

Written informed consent was obtained from the parents of the patient for publication of this case report and any accompanying images. A copy of the written consent is available for review by the Editor-in-Chief of this journal.

## Competing interests

The authors declare that they have no competing interests.

## Authors' contributions

GSS conceived and designed the study and wrote the initial draft of the manuscript. He acts as the guarantor. TM and SS collected the data and drafted the paper. GSS helped in the acquisition of significant intellectual content and the revision of the manuscript. The final manuscript was approved by all the authors.
